# The Contribution of Experimental *in vivo* Models to Understanding the Mechanisms of Adaptation to Mechanical Loading in Bone

**DOI:** 10.3389/fendo.2014.00154

**Published:** 2014-10-01

**Authors:** Lee B. Meakin, Joanna S. Price, Lance E. Lanyon

**Affiliations:** ^1^School of Veterinary Sciences, University of Bristol, Bristol, UK

**Keywords:** bone, mechanical loading, experimental models, mechanostat, mechanical strain

## Abstract

Changing loading regimens by natural means such as exercise, with or without interference such as osteotomy, has provided useful information on the structure:function relationship in bone tissue. However, the greatest precision in defining those aspects of the overall strain environment that influence modeling and remodeling behavior has been achieved by relating quantified changes in bone architecture to quantified changes in bones’ strain environment produced by direct, controlled artificial bone loading. Jiri Hert introduced the technique of artificial loading of bones *in vivo* with external devices in the 1960s using an electromechanical device to load rabbit tibiae through transfixing stainless steel pins. Quantifying natural bone strains during locomotion by attaching electrical resistance strain gages to bone surfaces was introduced by Lanyon, also in the 1960s. These studies in a variety of bones in a number of species demonstrated remarkable uniformity in the peak strains and maximum strain rates experienced. Experiments combining strain gage instrumentation with artificial loading in sheep, pigs, roosters, turkeys, rats, and mice has yielded significant insight into the control of strain-related adaptive (re)modeling. This diversity of approach has been largely superseded by non-invasive transcutaneous loading in rats and mice, which is now the model of choice for many studies. Together such studies have demonstrated that over the physiological strain range, bone’s mechanically adaptive processes are responsive to dynamic but not static strains; the size and nature of the adaptive response controlling bone mass is linearly related to the peak loads encountered; the strain-related response is preferentially sensitive to high strain rates and unresponsive to static ones; is most responsive to unusual strain distributions; is maximized by remarkably few strain cycles, and that these are most effective when interrupted by short periods of rest between them.

## Introduction

The effect of mechanical loading on bone has been studied since the nineteenth century. These studies have primarily utilized animal models of mechanical loading since bone adaptation is a highly complex process that is difficult to fully appreciate *in vitro* or *in silico*. The first aim of this review is to discuss the various models that have aided our understanding of this adaptive process. The second section will discuss the knowledge that has been gained from such *in vivo* studies, which have determined how to modify the mechanical strain stimulus to give a more osteogenic outcome. Finally, we will explore the effect of physiological context on bone’s adaptive response to loading using the example of estrogen’s role in the adaptive process and its contribution to post-menopausal osteoporosis.

## Wolff’s Law and the Mechanostat

In 1892, the anatomist and orthopedic surgeon Julius Wolff postulated that bone adapted to its mechanical environment according to strict mathematical laws. This principle, now commonly known as Wolff’s Law (1) was based on anatomical dissection studies which demonstrated increased bone mass in areas predicted to be subject to high mechanical stresses and low bone mass where stresses were predicted to be low. Incorporating previous work by the anatomist Meyer and the Swiss engineer Culmann, Wolff further observed that trabeculae in the femoral head and neck were orientated to reduce bending stresses when the material was loaded. These observations allowed Wolff to state his “Law” as “*Every change in form or function of bone or of their function alone is followed by certain definite changes in their internal architecture, and equally definite alteration in their external conformation, in accordance with mathematical laws*” (2). Wolff’s Law was expanded by Wilhelm Roux who suggested bone adaptation was the result of a “quantitative self-regulating mechanism” based in the tissue at the level of the cell (3).

Architectural bone changes such as those observed by Wolff were hypothesized to occur due to a dynamic adaptive process in response to changes in the mechanical stimulus derived from load-bearing. This theory was further developed by Harold Frost in 1960 whose thoughts form the basis of the current “mechanostat” theory of bone adaptation (4). Frost recognized that the most likely loading-derived stimulus for bone cells would be the strains developed in the bone tissue as a result of loading. His mechanostat theory suggests a negative feedback system in which the input is local mechanical strain, engendered by functional mechanical loading, and the output is a structurally appropriate bone mass and architecture. Accordingly, in areas of bone experiencing mechanical strains higher than the strain set point, bone formation would occur to make a stiffer bone construct thus reducing strains and restoring set point strains. Conversely, if bones experienced strains lower than the set point, then bone resorption would occur, bone mass would decrease and, for the same loads, strains would again be restored to the level of the set point. This theory was extensively described in the Utah Paradigm of Skeletal Physiology (4). It is an important component of this theory that the mechanostat is a local phenomenon with local changes in strain adjusting local bone mass through local bone formation or resorption. However, it is also important to remember that the mechanostat operates within a wider physiological context and that its ability to establish and maintain structural competence may be substantially modified, or overwhelmed, by systemic circumstances (5, 6).

## Extrinsic Models of Mechanical Loading

What neither Wolff nor Frost was able to state was the specific components of bone cells’ strain environment that act as the controlling stimulus for architectural modeling and remodeling. It is this question that has been addressed principally by animal experiments.

One of the simplest ways to increase the mechanical loads experienced by bones in the skeleton is to increase the level of exercise undertaken. A large variety of techniques have been described to achieve this in experimental animals, usually taking the form of forced exercise such as treadmill running (7–9), swimming (10–12), or jumping (13, 14). Other models have also been described in which animals (usually rats) wear increasingly weighted backpacks or have to press levers with increasing levels of resistance to get food rewards (15).

In these exercise models, the whole body is loaded through coordinated muscular contraction and ground reaction forces in a manner that is an exaggeration or extension of that to which they are habituated. Such studies are useful since they require little in the way of specialized equipment and they mimic the natural challenge to skeletal strength that the mechanostat presumably evolved to address. However, while exercise may be the easiest therapy to prescribe in an attempt to regulate bone mass or adjust bone architecture, it involves a mixed stimulus of change in loading and physiological context that makes the results difficult to interpret.

As well as (presumably) altering local mechanical strain, exercise engenders a great many other physiological responses such as increasing tissue blood flow and oxygenation (16, 17), changing the endocrine environment (18), muscular contraction, and local release of potentially osteogenic factors from muscle (19), which could complicate interpretation of the intrinsic loading-related response of the bone alone. A further disadvantage with exercise models is that exercise is a whole body phenomenon, therefore comparison has to be made with controls whose total exercise experience (both during the exercise periods and the rest of the day) has to mimic the experimental subjects in every respect, except the modified exercise under study. Lastly, but by no means least, the changes in the strain environment of bone cells brought about by change in exercise can usually only be estimated mathematically with no means of experimental verification. Such verification had to await the introduction of the chronically implanted electrical resistance strain gage (20).

## Quantifying the Mechanical Strain Stimulus

**Figure 1:**
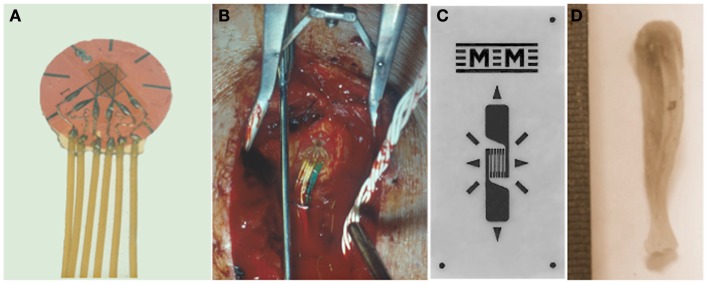
**Quantifying the mechanical strain stimulus**. Rosette **(A)** or linear **(C)** electrical resistance strain gages are bonded to the surface of bones to measure the strain stimulus being engendered during a variety of activities and also to match experimental groups with different bone stiffness. **(B)** A strain gage bonded to a human tibia and **(D)** to a mouse tibia.

Although Gaynor Evans had attached electrical resistance strain gages to bone surfaces *in vivo* in the 1940s (21), these were only used for the assessment of bone strains in response to impact loading in anesthetized animals in short term experiments. Lanyon and co-workers were the first to chronically implant electrical resistance strain gages attached to the surfaces of bones of a variety of living animals, including humans (Figure 1), and analyze the various components of the strain stimulus during a variety of activities (20, 22–30). In the majority of weight bearing long bones, the peak mechanical strains were remarkably similar across different species and activities measuring approximately 2–3000 με (2–3 × 10^−3^). Peak compressive strains have subsequently been recorded from the horse third metacarpal bone during galloping where the recorded maximum strain magnitude reached 5,000 με (5 × 10^−3^) (26).

This similarity in peak strains recorded from different bones from different species supported Frost’s mechanostat theory of bone adaptation and suggested that the set point for peak functional strain was similar across species. Nevertheless, it is important to remember that this similarity in peak strains across different load-bearing bones in different animals does not imply a single target strain at every skeletal location. Since the highest physiologically induced strains are due to bending, the peak longitudinal strains around the bone’s circumference will change from compression to tension and pass through the neutral axis. However, the cortical thickness is reasonably similar around the circumference of the bone. Additionally, significantly lower strains have also been recorded at other sites in load-bearing bones and on the surfaces of bones whose primary role may not be sustaining high loads. For example, low functional strains have been recorded on the skull during activities such as chewing, smiling, and even heading a ball (80–200 με). This suggests that different bones, and different sites on the same bone, have different local settings for the target strains that their mechanostat attempts to achieve (31).

Most of the early studies using bone-bonded strain gages were undertaken using rosette gages which respond to strain in three different directions and therefore allow the direction of the principal tensile, compressive, and shear strains to be calculated. These calculations confirmed the essence of Wolff’s hypothesis in his trajectorial theory that bone trabecule tend to be aligned in the directions of principal tension and compression strains (22). In addition to measurement of strain magnitude and direction, electrical resistance gages allow recording of the rate of change of strain (the strain rate) experienced by the bone during loading and unloading. The highest strain rate so far recorded during natural activity was from the sunfish operculum (the bone covering the gills) during striking at prey. At −615 × 10^8^ με this peak strain rate is over 10 times higher than anything so far recorded in terrestrial animals during locomotion (32). Despite the obvious advantage of using rosette strain gages they substantially complicate the experimental procedure due to their size and the extra number of channels that need to be monitored. Thus, particularly in studies on small animals such as rodents, it has been more usual to use single element gages (33, 34). These can only respond to strains in the direction in which they are aligned so cannot distinguish between changes in strain magnitude and changes in strain direction. When using single element gages it is important to ensure that the gage is aligned in the direction of interest and to realize that a reduction in strain reading may mean a change in direction as well as a reduction in strain magnitude.

Although used most widely, particularly *in vivo*, strain gages are not the only devices employed to measure mechanical strains on bone surfaces, particularly *ex vivo*. Other techniques include the use of digital image correction. This method requires a speckle pattern to be applied to the surface of the bone of interest. Sequential images are then taken during loading to trace the movement of the speckle pattern. This allows the resulting strain to be calculated across different areas of the bone surface (35). Finite element analysis is widely employed to calculate stresses (and thus strains) but this requires substantial assumptions to be made as to the load path within the bones and the material properties of the bone tissue, which are not homogeneous along the bone’s length (36, 37). Despite these limitations, finite element analysis allows estimation of the strain pattern throughout a whole bone, something that is impossible with strain gages, which can only sample the strain from the surfaces to which they are attached. Thus finite element analysis can extend knowledge of the strain situation to the depths of the bone and to the bone trabecule where strain measurements using strain gages would be impossible (38–40). Using finite element analysis combined with *in vivo* μCT scanning, accurate prediction of sites of bone formation and resorption following the application of mechanical loading has been possible (39).

Whichever technique is used to measure loading-engendered mechanical strains, it is vital that strains and strain rates are normalized between experimental groups. The same loads applied to bones of different size, shape, or material properties will inevitably result in different strains. Therefore the loads, and load rates, must be adjusted so that the strain stimulus is the same in all bones in the same study. It is only by doing this that their adaptive responses can be directly compared.

## Invasive Models of Mechanical Loading

While the availability of chronically implanted bone-bonded strain gages provided the means to sample the strain environment experienced by bone cells, it was still necessary to find a suitable means to alter this environment in a controlled manner and assess the adaptive response. One means used to achieve this was to surgically remove a bone, or piece of bone, where paired bones exist, to alter the functional strains on the remaining bone. An example of this technique is the ulna ostectomy experiments performed by Goodship et al. (41). In this experiment, a portion of the pig ulna was removed, which increased the strains during ambulation in the remaining radius. This had the effect of causing an increase in radial bone formation. After 3 months, the strains in the radius had normalized back to those measured in the contralateral radius in the intact limb. An advantage of this model over the whole body exercise models is that there is a control limb with which to compare bone’s adaptive response. However, osteotomy involves invasive surgery near to the site being investigated and thus it is only possible to monitor rather than control the various components of the strain stimulus.

**Figure 2:**
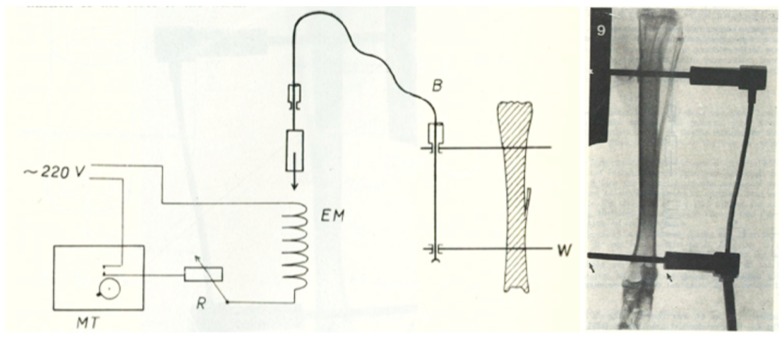
**The invasive rabbit loading model**. Jiri Hert was the first to describe an artificial mechanical loading model in 1969. Loading was applied through surgically implanted pins in the lapine tibia. Using this approach and assessing the adaptive response of the bone Hert and his co-workers were able to establish, amongst other findings, that adaptive bone (re)modeling occurred without the need for any central nervous connection (42–44).

**Figure 3:**
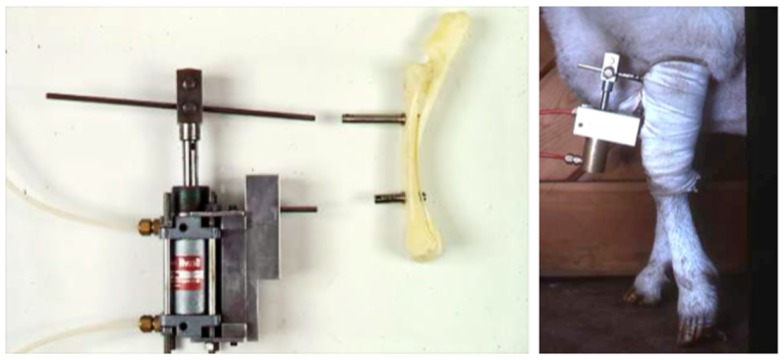
**The invasive sheep loading model**. Lance Lanyon adapted Hert’s original loading model to the sheep radius and combined the loading with strain measurements using implanted strain gages (45).

**Figure 4:**
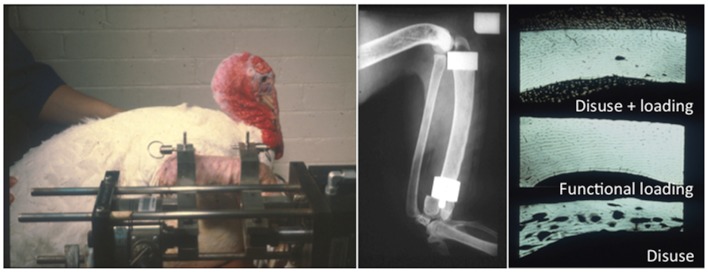
**The functionally isolated invasive turkey loading model**. Lanyon and Rubin further applied the invasive loading model to the functionally isolated avian ulna, which had the advantage that the loaded bone is isolated from the confounding influence of background strain stimuli (46–48).

The first successful attempts to control bones’ mechanical loading in order to study their adaptive responses was established in the 1960s by Jiri Hert who applied different loading regimens from an external electromagnetically operated device to the tibia of rabbits through transfixing pins (Figure 2). By using this approach and assessing the adaptive response of the bone Hert and his co-workers were able to establish, amongst other findings, that adaptive bone (re)modeling occurred without the need for any central nervous connection (42–44). Hert’s experiments, while ground breaking, lacked knowledge of what the actual strains were that his device engendered. This capability was established by Lanyon and co-workers by combining the techniques of loading a bone *in vivo* through transfixing pins with that of chronically implanted strain gages. Although the first animal they used for this approach was the sheep (Figure 3) (45) their most productive large animal model was the avian (rooster and later turkey) ulna (Figure 4) (46–48). This was chosen since the ulna was large enough to receive transfixing pins at its ends and three rosette strain gages around the circumference of its midshaft. Furthermore, as domestic turkeys and roosters are flightless, functional isolation of the bone’s natural loading did not greatly inconvenience their lifestyle.

The preparation used in the functionally isolated externally loadable avian ulna model involved performing a proximal and distal ulna osteotomy, placing metal caps over each end of the cut section of bone and securing them in place with metal pins placed through predrilled holes in the caps. This model has an advantage over the previous limb long bone models in that the section of bone being loaded is functionally isolated from the rest of the wing preventing any confounding strain stimulus derived from habitual activity. Using these models, several important observations were made documenting the effects of the different components of the strain stimulus on bone mass and architecture. These will be discussed in a later section of this review.

One of the disadvantages of the invasive loading models is that placing pins through the bone to be loaded invokes a local periosteal woven bone response (46, 48). This prevents the whole bone from being analyzed. Furthermore, since the majority of trabecular bone resides at either end of the long bones, this cannot be analyzed since it is not loaded. To overcome these problems, Chambers et al. developed the rat tail vertebra loading model (49). This involves placing pins through tail vertebrae in the rat (later adapted for the mouse) adjacent to the vertebra of interest that will undergo mechanical loading. This means that any periosteal woven bone reaction is limited to bones on either side of the one being loaded thereby permitting full analysis of the bone of interest. Furthermore, the tail vertebrae contain abundant trabecular bone enabling study of trabecular bone’s adaptive response to loading. For these reasons, the tail vertebra loading model is still in use today (50–52). However, there remains a concern that surgical placement of pins invokes not only the woven bone reaction in the region immediately surrounding the pin, but also a local inflammatory reaction, which could affect adjacent bones. Another concern is the relevance of bone adaptation in a bone that is not normally subject to such loads since it is likely that rodent tail vertebrae normally experience low strains during locomotion and other activities. A further disadvantage is that there must be a period, once the pins have been placed, to allow their integration into the bone prior to starting any loading studies.

## Non-Invasive Models of Mechanical Loading

The first of the non-invasive artificial loading models was described by Charles Turner et al. in 1991 (53) where he reported applying four-point bending to the rat tibia and observing the subsequent adaptive response. The loading arrangement consists of two contact points on the underside (the lateral aspect) of the limb and two on the upper medial side of the limb, which are inside those on the underside. A force is applied by downwards pressure from the upper contact points, which generates a bending of the tibia and causes a subsequent remodeling response. A sham loading arrangement where pressure is applied but no bending force generated can be created by placing the upper contact points directly opposite the lower ones. Although this model has advantages over the invasive models previously described, the contact points through which the loading is applied are quite close together and they apply their loads through the periosteum. Periosteal disturbance such as this is sufficient by itself to induce periosteal woven bone formation (54). This limited the region of analysis to the endosteal surface, which may behave inherently differently in response to strain from the periosteum.

To address some of the issues of the four-point bending model, Ted Gross’ group developed a cantilever bending model in which the knee and the ankle of the mouse hindlimb were placed in specially designed cups and the ankle moved laterally in relation to the knee to generate a bending moment in the tibia/fibula (55). This eliminated the periosteal woven bone observed with the previous bending model allowing analysis of both periosteal and endosteal bone compartments. However, there was still the concern that this bending moment does not accurately recreate the axial loading generated during ambulation in people and animals. Furthermore, only the central diaphyseal region of the bone displays a response to loading meaning the metaphyseal region containing the majority of the trabecular bone is not loaded and therefore does not display an adaptive process.

A significant advance was made when Torrance et al. (56) established that physiologically relevant strains could be engendered in the ulna of rats when their forearm was loaded axially through the skin over the olecranon and flexed carpus. They used the ulna rather than the tibia because the loads necessary to engender sufficiently high strains in the tibia caused necrosis of the skin over the knee and ankle. This disadvantage did not occur when the tibia was loaded in mice and these have more recently become the species of choice for such experiments (5, 33, 34, 57).

**Figure 5:**
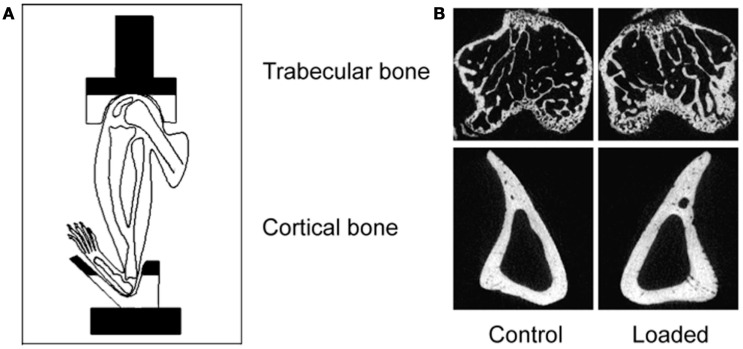
**The non-invasive axial murine tibial loading model**. **(A)** De Souza et al. adapted the non-invasive rat (56) and ulna (33) axial loading models to the tibia enabling study of trabecular and cortical compartments in a single loaded bone (34). **(B)** Representative μCT scans demonstrating trabecular and cortical bone formation within a single loaded bone (58).

The advantage of the non-invasive mouse tibial loading model is that precisely controlled loading regimens can be established in long bones in mice over a sufficient period of time to allow the adaptive response to be completed. Also no surgical invasion is required and the loading-related response throughout the whole bone can be studied. These substantial advantages were further increased when extended to the mouse fibula (57). The design of the mouse tibia/fibula loading model is relatively simple with custom-designed cups placed over the knee and tarsus (Figure 5). The uppermost cup is attached to the actuator arm of a hydraulic or electromagnetic materials testing machine and the lower cup attached to a load cell so that the load applied to the bone can be accurately controlled. Axial compression causes strain due to compression and bending of the bone increasing the strain stimulus and inciting an adaptive response.

Due to the placement of the cups in the tibia/fibula axial loading model, the whole bone is loaded including the metaphyseal region so the adaptive response can be observed in both cortical and trabecular bone compartments (5). Additionally, the loaded tibia and fibula have no direct contact with the loading machine cups since the load is applied through the distal femur and the flexed tarsus. Since this arrangement allows the simultaneous loading of both the tibia and fibula, the adaptive response in both bones can then be differentially processed for different analytical techniques reducing the number of experimental animals required. Finally, there are regions of the tibia (37% site measured from the proximal end) where the loading response is maximal while other regions (75%) where no loading response is observed (5). Interestingly, maximal strains are estimated to be similar at both regions however, down-regulation of osteocytic sclerostin, a necessary step in bone’s response to mechanical loading (59), is more closely related to sites of bone formation than to maximum strain (36).

Because the axial loading models can be used in both the rat and mouse, it is possible to use genetically modified animals enabling the role of specific genes (and pathways) in bone’s adaptive response to be determined (60–62). One of the disadvantages with the axial loading models is that the bone is stiffer when loaded in this direction, compared to being loaded in a non-physiological direction, so the loads required to engender an adaptive response are higher in the axial models than the bending models. A consequence of this may be premature closure of the growth plates when skeletally immature animals undergo axial loading of the ulna or tibia and fibula (63, 64).

## Models of Mechanical Unloading

In addition to studying the effect of artificially increasing mechanical strain and observing how bone adapts to this change in strain, it is also important to determine how bone adapts to a decrease in the mechanical strain stimulus. As well as increasing our knowledge of both extremes of the mechanostat theory of bone (re)modeling, these models can also be used to simulate clinical conditions associated with low bone mass such as disuse osteoporosis and bone loss following space flight.

The ideal model would allow bone strains to be reduced to zero. However, even in animals experiencing weightlessness during space flight, muscle contractions will still generate strains on the bone surface. Nevertheless, multiple animal studies have been performed during space flight and the resultant bone resorption has been studied (65–68). However, for most experiments this is not a feasible option.

More practical alternative experimental models of mechanical unloading have been described in the literature, which include applying a cast to a limb, performing an Achilles tenotomy to unload the calcaneus, performing a tenotomy of the knee tendons to unload the tibia, and bandaging a limb to the abdomen (69–75).

A surrogate to recapitulate the conditions of weightlessness is the tail suspension model (76). Using this model, mice or rats have their hindlimbs suspended by their tail while they are free to move around “normally” on their forelimbs. This model eliminates ground reaction forces on the hindlimbs although again, muscular contractions will still produce some mechanical strain on the bones involved. Using this model, researchers have demonstrated marked reductions in bone mass and deterioration in bone architecture (77–79). An adaptation of the tail suspension model has been developed, which allows graded levels of mechanical unloading (80). To try and further recapitulate the effects of space flight, one group simultaneously irradiated the tibia of mice and then performed hindlimb suspension (81). Interestingly, the effect of mechanical unloading on bone mass combined with radiation was far more extensive than that of radiation alone. Although hindlimb suspension causes measureable bone loss in the tibia, several disadvantages are associated with this model. Firstly, special cages are required, which have pulley systems to allow the animals to move around the cages. Secondly, there is a redistribution of blood to the cranial end of the animal with some systemic stress responses observed (82). Thirdly, this model causes bilateral bone loss losing the ability to compare one limb with its contralateral control and thus at least doubling the number of experimental animals required. Finally, in some countries, e.g., the UK, this model has been prohibited based on concerns for animal welfare since animals are unable to move freely.

A further two methods have been reported, which induce neurogenic paralysis of the hindlimb and therefore bone loss. This is achieved either by sciatic or tibial denervation or by the injection of botulinum toxin (83–86). These neurogenic paralysis methods have the advantage of reducing bone surface strains to around 300 με (one tenth of the normal peak strains) and the tibia can be subsequently reloaded using the axial tibial loading model to prevent bone loss (85, 86). Both approaches have their advantages and disadvantages. Sciatic neurectomy causes a permanent paralysis whereas botulinum toxin only lasts 1–2 weeks before some ambulation is regained. Sciatic neurectomy causes a more complete paralysis since botulinum toxin injection-induced paralysis, which affects the neuromuscular junction, relies on separate injections into each muscle group. Finally, botulinum toxin is more difficult to obtain due to its status as a prescription only medicine. In contrast, sciatic neurectomy requires minimal surgical instruments and the surgery is simple taking approximately 3–4 min per mouse in experienced hands.

A recent paper has reported the combined effects of hindlimb suspension and injection of botulism toxin (87). This showed that bone and muscle loss following hindlimb suspension and botulinum toxin injection were similar but when combined had additive effects. Interestingly, they also report bone and muscle loss in the contralateral (non-injected) hindlimb following botulinum toxin injections alone, suggesting systemic effects even though the injection primarily targets local muscle. An additional consideration is that inducing unilateral paralysis may increase load-bearing in the contralateral limb, although this does not appear to hold true for the botulinum toxin model.

## Knowledge Gained from *In vivo* Animal Loading Models

### Bone responds to dynamic but not static loads

Hert was the first to publish data suggesting that bone (re)modeling was not affected by static strains but rather by dynamic strain change (42). This inference was confirmed by one of the first studies published using the isolated avian ulna loading model. This study compared the effect of a continuous static load with that of the same magnitude of load applied dynamically for a single 100-s period per day in a trapezoidal waveform for an 8-week period (48). When the load was applied in a static manner, there was a 13% loss of bone due to endosteal expansion and an increase in cortical porosity. This level of bone loss was no different to that exhibited by the control group, which underwent osteotomy but no loading. However, the group in which the load was applied in a dynamic manner exhibited a 24% increase in bone mass due to periosteal bone formation. For this reason, the majority of studies using exogenously applied load apply a dynamic waveform of either triangular, sinusoidal, or trapezoidal shape (5, 34, 38, 88–90). However, to our knowledge, no studies have directly compared the relative effects of these different waveforms on bone formation.

### Loading-related bone formation correlates with peak strain magnitude

**Figure 6:**
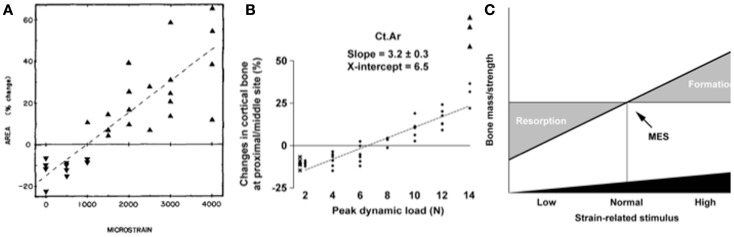
**The “lazy zone” is an artifact associated with background mechanical strain stimuli**. Increasing mechanical strain is associated with a linear increase in bone mass in studies when background strains are eliminated in the isolated avian ulna [**(A)**, (91)] and combined sciatic neurectomy and tibial loading models [**(B)**, (86)]. This indicates there is no “lazy zone” in bone’s adaptation to loading [**(C)**, (86)]. MES, minimum effective strain stimulus.

The single most important component of the strain stimulus to engender an osteogenic response to mechanical loading is the peak strain magnitude. Multiple studies by our laboratory and others have demonstrated that by varying the peak strain magnitude, but keeping all other parameters the same, there is a dose:response relationship between the peak strains engendered and the amount of new bone formed (53, 61, 86, 88, 91). Several previous studies made the assumption that there was an adapted window or lazy zone in which bone mass and architecture would not change over a set range of strain stimuli. However, in the isolated turkey ulna where disuse and artificial loading are combined, there was no evidence of this window (91). A more recent study, stimulated by editors’ refusal to accept the veracity of this earlier finding, confirmed the absence of this lazy zone using the combined sciatic neurectomy and axial tibial loading model (Figure 6) (86). This is also supported by the graded mechanical unloading model of Ellman et al., which confirmed a linear relationship between percentage weight bearing and bone loss (80). It is important to realize that these experiments refer only to a single loading modality. In the natural situation where the osteoregulatory effects of all the normal loading modalities are added together this may constitute a spectrum of strain magnitudes that averaged within a particular window do indeed result in little or no adaptive response.

As previously discussed, the mechanostat is a local phenomenon (5, 6, 92). As such, mechanical loading will engender a range of peak strain magnitudes in different regions of the same bone (34, 36). Studies have repeatedly shown periosteal bone formation following mechanical loading to occur primarily in regions where strains are high and lower levels of bone formation, or even resorption, to occur where peak strains are lower (37, 51, 93, 94).

### Strain-related stimulus is determined by the novelty of loading

In the avian ulna studies, substantial increases in bone mass occurred with artificially applied loads, despite these loads engendering lower peak maximum strains and strain rates than those measured during normal wing flapping (46). Thus it is impossible to say that peak strain *per se* engendered the strain-related stimulus. It is instead likely that this substantial stimulus arises from the strains being engendered in a novel configuration. Similarly, in the tibial bending studies (53, 55), relatively low loads were required to induce adaptive new bone formation. Conversely in the axial loading models where the load is being applied more closely to the normal direction in which the bone is loaded, higher loads appear to be necessary to generate new bone formation, although since the bone is more resistant to load in this direction the strains may be similar in magnitude.

Nevertheless, a recent study used different enclosure designs to force growing female C57Bl/6J mice to use non-linear, diverse-orientation locomotor loading showed that, when compared to mice using primarily linear locomotion, those with diverse non-linear locomotion had increased humeral trabecular bone volume fraction and indices of cortical bone volume (95). This is despite no difference in overall activity levels between mice in the two different types of enclosure. These findings support a previous study that used transarticular external fixators in sheep to prevent calcaneal load-bearing, which resulted in bone loss (96). When disuse was interrupted by periods of steady walking on a treadmill, when the tarsus was mobilized, the strain stimulus from walking was unable to prevent bone loss suggesting either that the duration of the stimulus was insufficient [unlikely in view of the turkey ulna data (47)] or that the variety or novelty of the strain-related stimulus was not being provided. Another study demonstrated that fighting between male mice housed in groups was associated with an increase in measures of cortical and trabecular bone mass and architecture, which “masked” the effect of mechanical loading in these animals (97). When males were individually housed, and thus not involved in vigorous diverse activity, their response to mechanical loading was no different from that observed in group or individually housed female mice, which did not fight.

### Increasing strain rate during loading and unloading stimulates bone formation

The rate of change of strain magnitude, both when load is applied and released, is an important determinant of a strain regimen’s osteogenic potential. This was first suggested in an early study using an invasive loading model in sheep where (in the rather uncontrolled loading situation achievable with a pneumatic loading device) change in strain rate accounted for more of the variation in the amount of new bone formed in response to loading than peak strain magnitude (45). Interestingly, this effect of maximum strain rate was unaffected by whether this occurred during loading (compression) or unloading (tension). A later study using the much better controlled rat ulnar axial loading model demonstrated, using various strain rates but the same peak magnitude, number of loading cycles, etc. that increasing the strain rate by approximately fivefold (from −0.018 to −0.100 s^−1^) increased the osteogenic bone formation response both in amount and extent (98). A further study by Turner et al. found similar results concluding that the amount of new bone formation was directly proportional to the rate of change in strain in bone tissue (99). However, one of the difficulties with interpretation of both of these studies is that to increase strain rates but keep all other parameters the same, a period of rest has to be inserted between each of the loading cycles. Although not appreciated at the time there is now evidence that such a rest period can itself enhance bone’s adaptive response to artificial loading. Despite this reservation, the consensus from a number of studies is that the rate of change of strain is an important determinant of the stimulus for a bone formation response. This finding is consistent with the results from human studies; exercise regimens involving high levels of impact loading (squash, tennis, and triple jump) being far more effective than those that do not (swimming and cycling) in stimulating new bone and thus increasing bone mass (100–103). In an additional study by LaMothe et al. using the cantilever bending model, they increased the dwell time (the length of time the load was applied for) when the strain rate was increased ensuring the duration of each loading cycle and frequency of cycles was consistent between groups (104). Again they showed a significant increase in the bone formation response when strain rate was increased.

In the first study describing the non-invasive axial tibial loading model, there was actually a loss of trabecular bone volume fraction reported, which we now believe is due to their use of a relatively low strain rate of 1,500–2,000 μεs^−1^ during loading and unloading (34). Another more recent paper using a much higher number of cycles but with the same low strain rate reported in the first study also documented a loss of trabecular bone in the proximal tibia with loading (38). However, studies using an identical experimental protocol, but with a strain rate 10 times higher than that used in the first study, have consistently reported a robust increase in trabecular bone volume fraction, thickness, and even number (58, 61, 62, 86, 97, 105). To the authors’ knowledge, no studies have directly compared the influence of strain rate on the relative responses of cortical and trabecular bone.

### Number of loading cycles required to maximally stimulate bone formation is small

Two important studies have compared the number of cycles of loading required to stimulate an osteogenic response and have shown it to be surprisingly small. Rubin and Lanyon first demonstrated in the isolated avian ulna that increasing the number of loading cycles in a given episode of mechanical loading from 36 to as many as 1,800 did not generate any additional adaptive response (46). Perhaps as interesting, was their finding that only four loading cycles a day were sufficient to prevent the bone loss associated with disuse (zero cycles). Another study in rats trained to jump a 40 cm height showed little additional effect on increasing the number of jumps from 5 up to 40 per day (14).

Although relatively few cycles of loading are required to maximize the amount of new bone formed, there are a number of publications using *in vivo* mechanical loading where several thousand loading cycles are performed. For some this is a deliberate design to induce fatigue loading of bone and examine subsequent damage repair (106). However, for many studies, although a robust way of ensuring osteogenesis, this high number of cycles is likely to be unnecessary and may indeed have detrimental effects. These include an increase in the length of anesthesia time required for each episode of loading and also an increased likelihood of bone and soft tissue damage.

### Inserting rest between loading cycles increases bone formation

A study by Robling et al. demonstrated that partitioning 360 loading cycles into 4 bouts of 90 cycles or 6 bouts of 60 cycles per day enhanced the osteogenic response to loading (107). Robling went on to demonstrate that inserting a 14-s rest period between loading cycles optimized the bone formation response to loading (108). This was confirmed by LaMothe and Zernicke using the cantilever bending model which showed that when a 10 s rest period was inserted every 11 s in a 30 Hz 100 s loading protocol, the bone formation response increased by 72% despite a 100-fold decrease in the number of loading cycles (104). Additional studies by Gross’ group, also using the cantilever bending model, observed that inserting a 10 or 15 s rest period between each loading cycle enhanced the osteogenic stimulus in both young and aged mice (109–111). Finally, a study by Charles Turner’s group demonstrated that as the length of the loading study is increased, the percentage new bone formation response per week decreases suggesting the stimulus may become less potent over time. However, introducing a rest period of several weeks between bouts of mechanical loading can partially restored this decreased response to loading (112).

## Maximizing the Strain Stimulus

It is important when designing a loading protocol that all of the evidence reported in the previous section is taken into account. The method for applying mechanical load using the tibial loading model has already been extensively described (34, 113). The protocol in current use in our laboratory is as follows:

The tibia is held in place by a 0.5 N continuous static pre-load. Forty cycles of dynamic load are superimposed using a trapezoidal waveform with 10 s rest intervals between each cycle. The protocol for one cycle consists of loading to the target peak load, hold for 0.05 s at the peak load, and unloading back to the 0.5 N pre-load. Strain gaging data from our current materials testing machine (Bose ElectroForce 3100) indicate that a load of 15N is equivalent to 2500 με on the medial surface of the tibia at 37% of the length from the proximal end in a young adult 17-week old female C57Bl/6J mouse. The load rate we use in young adult female mice is 500Ns^−1^, which equates to an average strain rate of 30,000 μεs^−1^. These values are within the normal physiological range recorded for this species (34). Using this protocol, we observe approximately a 20–30% increase in cortical bone area at the 37% site and approximately a 50–75% increase in trabecular BV/TV in the secondary spongiosa. While we do not believe that the same loading protocol should be recapitulated in all laboratories using the non-invasive axial tibial loading model, designing a protocol should be based on previous studies so that interpretation of novel or unexpected results is made more reliable.

## Influence of Physiological Context on the Mechanostat

As discussed earlier in this review, the mechanostat is primarily a local phenomenon in which bone responds to local changes in mechanical strain and adapts its local bone mass and architecture accordingly to adjust strains to restore the set point. However, it is well accepted that the functioning of the mechanostat can be influenced by systemic changes. For example, during the menopause in women there is a loss of bone mass sufficient to increase the incidence of fragility fractures (114, 115). The rapidity of this loss is unlikely to be accounted for by any sudden change in mechanical loading of the bone through decreased exercise, etc. Therefore, there must be some mechanism, which influences the mechanostat and impairs its function (116). The most likely candidate is decline in the levels of available estrogen and we have attributed at least part of this failure to altered function of its receptors; estrogen receptor (ER) α and β (62, 90). Aspects of the changes that are likely to occur with the menopause have been recapitulated in animal models where loading can be artificially increased and therefore allowing interrogation of the link between the mechanostat and estrogen receptor signaling in more detail. The interventions used to investigate this mechanism will now be discussed.

### Surgical modeling of pathology

A simple way to recreate the menopause and deplete endogenous estrogen in animals is by removal of the gonads i.e., an ovariectomy. The earliest study which investigated the effect of ovariectomy on the loading response was using the four-point bending model in rats (117). They concluded that the cortical response to loading was not affected by ovariectomy, although a concurrent loss of trabecular bone due to estrogen depletion was observed. As discussed earlier in this review, there are issues, which surround the use of bending models of loading, particularly in these circumstances where analysis of trabecular bone is key. However, more recently the lack of the effect of ovariectomy on the adaptive response has been confirmed using the axial tibial loading model (60). Chambers’ group have also investigated the effect of combining ovariectomy with invasive mechanical loading of the rat tail vertebra, which contains predominantly trabecular bone (118). Unexpectedly, they found that the trabecular bone formation rate following loading was increased in ovariectomized compared to control animals. Interestingly, the enhancing effect of ovariectomy on the response to loading was not inhibited by the addition of pamidronate, a bisphosphonate, which inhibits bone resorption by inducing osteoclast apoptosis. This suggests that estrogen may have an inhibitory effect on osteoblastic bone formation such that once removed by ovariectomy, loading-induced bone formation is increased. More recently, these findings have been confirmed using the non-invasive axial tibial loading model where it was also demonstrated that ovariectomy increases the loading-related gain in trabecular but not cortical bone (119).

### Pharmacologic interventions

In addition to investigating the effect of loss of estrogen following ovariectomy on the function of the mechanostat, studies have also been performed to examine the effect of estrogen supplementation by injecting rats daily with biologically active 17β-estradiol (118). In agreement with the previously mentioned enhanced response to loading following ovariectomy, in trabecular bone, estrogen supplementation inhibited the response to loading. This was also unexpected since estradiol is well accepted to prevent the deleterious effects of ovariectomy on trabecular bone (120). Interestingly, estrogen also appears to have an inhibitory effect on cortical bone’s response to mechanical loading (121), which is surprising given the lack of effect of ovariectomy on cortical bone’s response to mechanical loading (60, 119). One of the complexities encountered when interpreting these studies are the relative effects of ERα and β activation. Newer drugs have been developed, which act as selective ER modulators (SERMs). One of these SERMs, tamoxifen, is used clinically as a breast cancer treatment and has been shown to be more potent than estradiol at preventing the ovariectomy-induced loss of trabecular bone (120). Perhaps more beneficially, it has been demonstrated that tamoxifen causes a synergistic increase in trabecular bone formation when combined with mechanical loading (119).

### Genetic manipulation

To further decipher the relative involvement of ERα and β in bone’s response to mechanical loading, an alternative to the administration of selective pharmacologic agents is to create animals, usually mice, with genetic modifications and apply mechanical loading. As discussed previously, one vital undertaking prior to mechanical loading of genetically modified animals is to determine whether the intrinsic load:strain relationship in these animals has also been modified such that similar strain stimuli can be applied to mice with potentially different bone phenotypes. The first study to investigate the effect of ER deletion on bone’s response to mechanical loading was by Lee et al. (90). Using the axial ulna loading model (33), they demonstrated that global deletion of ERα in mice significantly impaired cortical bone’s adaptive response to mechanical loading. Later studies confirmed these findings using the axial tibial loading model (60, 62, 122). One of these studies subsequently investigated the effect of genetic modifications of the specific activation function regions of the ERα found that deletion of AF1, but not AF2, was responsible for the effect of ERα knockout on the cortical response to mechanical loading (60). This is interesting because estrogen’s effect on bone mass is via AF2 and not AF1 (123) suggesting that in cortical bone, the effect of mechanical loading is via the ERα but independent of estrogen itself, potentially explaining why ERα deletion, but not depletion of estrogen, decreases bone’s adaptive response to mechanical loading.

Other studies have investigated the role of ERβ using the non-invasive ulna and tibia loading models and found that global deletion of ERβ in mice enhances cortical bone’s response to mechanical loading (62, 124). Taken together, these data suggest that ERα and β have opposing roles in bone’s adaptation to loading. However, the story is more complex since global deletion of one ER can result in up regulation of circulating estrogen and other hormones confounding interpretation of these experiments (125–127). With the more recent advent of cell-type specific knockout mice being available (128–130), the cell-type specific role of ERs in the mechanostat is currently being further investigated. For example, deletion of ERα specifically from osteocytes has no effect on cortical bone’s adaptation to loading (128).

Another level of complexity is added when the role of the ERs in trabecular bone’s adaptation to loading is considered. In female mice, deletion of ERα does not affect the response to loading while in male mice ERα deletion increases the response to loading (62). In contrast deletion of ERβ enhances loading-related trabecular bone gain (62). However, interpretation of the effect of ER deletion on loading-related changes in trabecular bone is problematic since strain-matching between groups is not possible due to the limited means to measure loading-engendered strains in these compartments and because deletion of ERα, and ERβ in female but not male mice, increases trabecular bone mass (131).

Inevitably, it is to be expected that elucidation of the role of estrogen and the ERs is likely to be complex and to complete our understanding it is likely that a combined *in vivo* and *in vitro* approach will be required. However, the purpose of its discussion here was to illustrate the type of interventions that can be combined with mechanical loading studies to determine the mechanisms involved in the mechanostat in more detail.

## Conclusion

In this review, we have discussed the animal models of mechanical loading, which are available to dissect the mechanisms of the mechanostat and how they have been used to determine the components of the strain stimulus involved in bone’s adaptive response to mechanical loading. The complex process of bone modeling and remodeling can only be fully observed in animals and humans. Nearly all the understanding we have of the bones’ adaptive response to mechanical loading has been derived from experiments using animal models.

In summary, over the physiological strain range bones’ mechanically adaptive processes are responsive to dynamic but not static strains; the size and nature of the adaptive response is linearly related to the peak strains engendered; it is preferentially sensitive to high strain rates and unresponsive to static ones; it is most responsive to unusual strain distributions; it is maximized by remarkably few strain cycles, and that these are most effective when interrupted by short periods of rest between them.

No studies have so far demonstrated any unique mechanically responsive pathway in bone cells, rather the mechanisms of the mechanostat appear to be those used by a wide variety of cells to also respond to endocrine, paracrine, and autocrine influences. How these complex influences combine to so precisely regulate bone architecture that in the vast majority of situations it is appropriate to each individual’s changing loading history remains to be determined. When it is, much of the essential data contributing to the solution will have been generated by animal experimentation of the type described here.

## Conflict of Interest Statement

The authors declare that the research was conducted in the absence of any commercial or financial relationships that could be construed as a potential conflict of interest. The Specialty Chief Editor Jonathan H. Tobias declares that, despite being affiliated to the same institution as the authors, the review process was handled objectively and no conflict of interest exists.
